# Influences of calcium silicate on chemical forms and subcellular distribution of cadmium in *Amaranthus hypochondriacus* L.

**DOI:** 10.1038/srep40583

**Published:** 2017-01-11

**Authors:** Huanping Lu, Zhian Li, Jingtao Wu, Yong Shen, Yingwen Li, Bi Zou, Yetao Tang, Ping Zhuang

**Affiliations:** 1 Guangdong Ecological Meteorology Center, Guangzhou 510080, PR China; 2 Key Laboratory of Vegetation Restoration and Management of Degraded Ecosystems, South China Botanical Garden, Chinese Academy of Sciences, Guangzhou 510650, PR China; 3 Department of Ecology, School of Life Sciences/State Key Laboratory of Biocontrol, Sun Yat-sen University, Guangzhou 510275, PR China; 4 School of Environmental Science and Engineering, Sun Yat-sen University, Guangzhou 510275, PR China

## Abstract

A pot experiment was conducted to investigate the effects of calcium silicate (CS) on the subcellular distribution and chemical forms of cadmium (Cd) in grain amaranths (*Amaranthus hypochondriacus* L. Cv. ‘K112’) grown in a Cd contaminated soil. Results showed that the dry weight and the photosynthetic pigments contents in grain amaranths increased significantly with the increasing doses of CS treatments, with the highest value found for the treatment of CS3 (1.65 g/kg). Compared with the control, application of CS4 (3.31 g/kg) significantly reduced Cd concentrations in the roots, stems and leaves of grain amaranths by 68%, 87% and 89%, respectively. At subcellular level, CS treatment resulted in redistribution of Cd, higher percentages of Cd in the chloroplast and soluble fractions in leaves of grain amaranths were found, while lower proportions of Cd were located at the cell wall of the leaves. The application of CS enhanced the proportions of pectate and protein integrated forms of Cd and decreased the percentages of water soluble Cd potentially associated with toxicity in grain amaranths. Changes of free Cd ions into inactive forms sequestered in subcellular compartments may indicate an important mechanism of CS for alleviating Cd toxicity and accumulation in plants.

Cadmium (Cd), one of the most typical deleterious metals widely present in farmland soils, has the features of high toxicity, mobility and bioavailability^1^,^2^. Cadmium in soils is easily absorbed by plants^3^. It is suggested that the critical leaf concentration for toxicity of Cd is 5–10 mg/kg (based on dry mass) for most of plants^4^. Cadmium in plants may easily cause physiological, biochemical, morphological and structural changes in growing plants ultimately leading to reduction in productivity^5^.

To mitigate the problem of Cd uptake by plants, especially by food crops, remediation approaches involving Cd immobilization in soil are receiving increasing attention. Some nontoxic amendments have been added into soils to reduce the mobility and availability of Cd in soils through precipitation, adsorption or complexation with organic matters^6^. Several siliceous materials have been proven to be beneficial in mitigating Cd toxicity or reduction of Cd accumulation of many plants (either monocotyledonous^7^,^8^ or dicotyledonous^9^,^10^). In our previous research, we found calcium silicate (CS) is one of the promising potential candidate amendments for reducing heavy metal accumulation, providing an alternative immobilization remediation technique for soils polluted by heavy metals. We have noticed that the application of CS can lead to a redistribution of Cd to less mobile forms and reduce Cd uptake by plants due to its effect on soil pH regulation^10^. The liming effects of CS promote negative charging of soil surface, leading to an increase of adsorption capacity for Cd^11^. Monosilicic acid generated as a result of the H^+^ neutralization ability of silicate anion could complex with heavy metals in the soil solution to form slightly soluble or insoluble metal compounds of silicates^12^. In addition to the positive effect of silicate ion in soil, CS also supply calcium which is an important nutrient for plants growth and reduce plants Cd uptake by competing ion channels with Cd^13^,^14^. However, there is still not sufficient evidence to clarify the mechanism of inhibitory effect of CS on Cd accumulation in plants^15^. How CS might affect heavy metal distribution and detoxification in plants remains uncertain, like the modification of the subcellular distribution and chemical forms of Cd in plants.

In general, changes in subcellular distribution and chemical forms of heavy metals were proven to be closely linked to metal accumulation and tolerance in plants^16^,^17^. Weng *et al*.^18^ reported that most Cd was localized in the cell wall and the lowest amount was in the membrane of *Kandelia obovata*. In the cases of lettuce^19^ and ramie^16^, large fractions of Cd were found in the cell wall fraction. It has been documented that inorganic and water-soluble organic Cd, which can be extracted by 80% ethanol and deionized water respectively, has greater likelihood of migration than the other extracted forms and metals binding to pectates, phosphates, oxalates and residuals are less toxic to plants^16^,^20^.

*Amaranthus hypochondriacus* L. is a source of food in many temperate and tropical countries, and also is cultivated as a high quality forage or silage crop with abundant nutrition^21^, and is found to be capable of accumulation of high concentration of Cd in our previous study^22^. Studying the effects of amendments on Cd uptake by this widely consumed crop cultivated on heavy metals contaminated soil is of great importance for improvement of soil remediation technology.

Therefore, in the present study, we investigate the effects of calcium silicate on the growth and subcellular Cd accumulation of grain amaranths grown in Cd contaminated soil. We also examined the changes of chemical forms of Cd in grain amaranths with different CS addition dosages. Our results elucidate the possible mechanism of CS on Cd uptake and resistance in grain amaranths, and provide new insights into the efficiency of CS-induced immobilization of heavy metals in soils.

## Results

### Plant Growth and Chlorophyll contents

**Figure 1:**
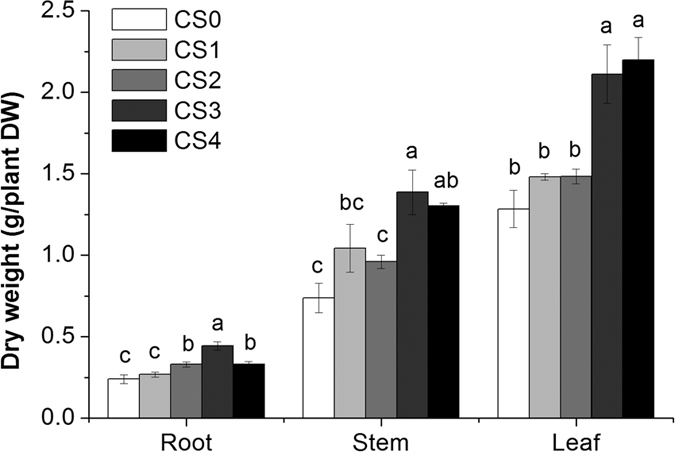
Effect of calcium silicate on dry weight of grain amaranths grown in Cd contaminated soil. Five treatments were used with calcium silicate added at various doses (0 (CS0), 0.41 (CS1), 0.83 (CS2), 1.65 (CS3) and 3.31 (CS4) g/kg). Error bars represent +/− SE of the quadruplicates. The means marked with the same letter at the same stage are not significantly different (*p* > 0.05).

The biomass of grain amaranths increased with the increasing level of silicate application, reaching the maximum values under CS3 treatment (Fig. 1). Compared to the control (CS0), an addition of 1.65 g/kg CS (CS3) to soil increased the dry weights of roots, stems and leaves of grain amaranths by 85%, 88% and 64%, respectively. However, there was no further growth promoting effect from increasing the CS addition from 1.65 to 3.31 g/kg (CS4).

**Figure 2:**
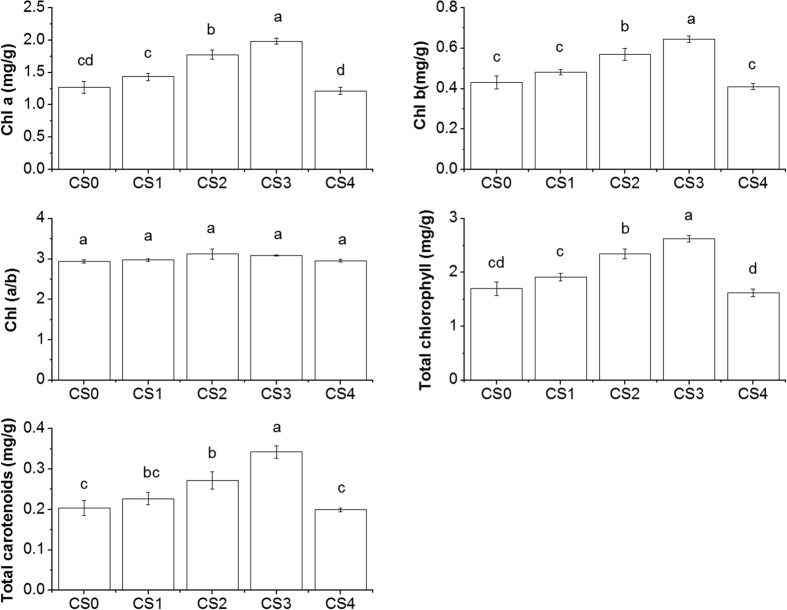
Effect of calcium silicate on the chlorophyll a (Chl a), chlorophyll b (Chl b), Chlorophyll (a/b), total carotenoids (Car) and total chlorophyll (Chl (a + b)) in the upper fifth leaves of grain amaranths grown in Cd contaminated soil. Five treatments were used with calcium silicate added at various doses (0 (CS0), 0.41 (CS1), 0.83 (CS2), 1.65 (CS3) and 3.31 (CS4) g/kg). Error bars represent +/− SE of the quadruplicates. The means marked with the same letter are not significantly different (*p* > 0.05).

Application of CS greatly influenced the chlorophyll contents with the highest chlorophyll contents in CS3 treated plants (Fig. 2). By addition of 1.65 g/kg CS into soil, the Chl a, Chl b, Chl (a + b) and Car contents in leaves of grain amaranths were 56%, 49%, 68% and 55% higher than that of the control plants, respectively. However, there was no significant difference in chlorophyll value of grain amaranths between the highest rate of CS4 and the control. Unlike the change of pigment contents, different CS treatments did not showed statistically significant effects on Chl (a/b).

### Concentration of Cd

**Figure 3:**
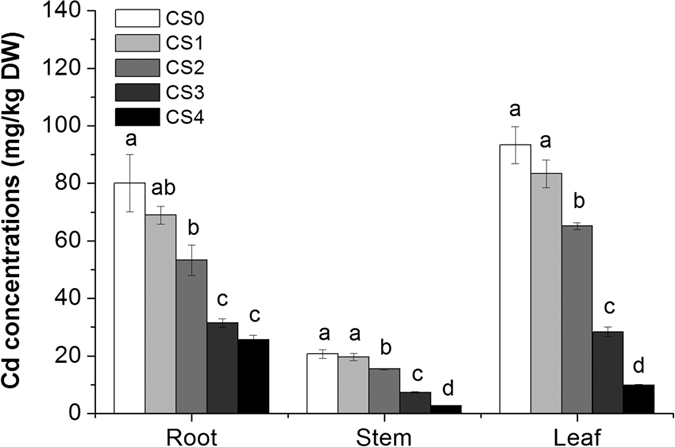
Cadmium concentrations in roots, stems and leaves of grain amaranths grown in a Cd contaminated soil treated with increasing doses of calcium silicate (0 (CS0), 0.41 (CS1), 0.83 (CS2), 1.65 (CS3) and 3.31 (CS4) g/kg). Error bars represent +/− SE of the quadruplicates. The means marked with the same letter at the same stage are not significantly different (*p* > 0.05).

Cadmium concentrations in grain amaranths decreased with the increasing doses of CS application, with the lowest value at the rate of 3.31 g/kg CS (Fig. 3). Cadmium concentrations in roots, stems and leaves of grain amaranths in CS4 treatment were 68%, 87% and 89%, respectively, lower than that of the control.

### Subcellular distribution of Cd

Table 1 summarized the subcellular distribution of Cd in leaves and roots of grain amaranths. The results showed that a majority of the Cd was located in the cell wall and soluble fractions. Partitioning of Cd among all fractions in leaves followed the pattern of: soluble fraction (FIV, 52–64%) > cell wall (FI, 24–39%) > chloroplast (FII, 6.4–12%) > membrane and organelle containing fraction (FIII, 2.6–4.2%), while in roots, the order changed slightly to cell wall (FI, 38–44%) > soluble faction (FIV, 28–37%) > trophoplast (FII, 16–22%) > membrane and organelle containing fraction (FIII, 6.0–7.1%).

Application of CS resulted in redistribution of Cd in grain amaranths. In leaves, the percentage of cell-wall-bound Cd decreased due to CS addition, while those of chloroplast and soluble fractions showed a tendency to increase. Especially for cell wall fraction, the percentage of Cd significantly decreased from 39% (CS0) to 24% (CS3). However, the percentage distribution of Cd in subcellular root tissues was not significantly different between treatments.

### Chemical forms of Cd

Cadmium concentrations of different chemical forms in grain amaranths decreased with the increasing levels of CS addition (Table 2). The predominant forms of Cd were extracted by 1 M NaCl in both leaves and roots. In leaves, the average proportion of the 1 M NaCl extractable Cd was 56% of the total Cd amount, followed by 2% HAC and d-H_2_O, with the residual forms of Cd being the lowest. In roots, Cd extracted by 1 M NaCl accounted for 82% of the total Cd, followed by that of d-H_2_O, and the residues had the lowest Cd.

The application of CS changed the percentage composition of Cd chemical forms in grain amaranths by elevating the proportion of 1 M NaCl, 80% ethanol, 0.6 M HCl extractable Cd and the residual form in both leaves and roots, but lowering those of the forms extracted by d-H_2_O and by HAC. Especially in leaves of CS4 treated plants, significantly increased percentages of 1 M NaCl (from 46% to 67%) and 80% ethanol (from 2.6% to 6.5%) extractable Cd, as well as slight increases in those of 0.6 MHCl extracted and residual forms, were observed, while remarkable decreased proportions of d-H_2_O (from 21% to 5.0%) and HAC (from 28% to 19%) extractable forms of Cd were found.

## Discussion

Calcium silicate (CS) is known as an agricultural liming material usually used to regulate soil physical, chemical and biological properties^23^. Studies have now shown the beneficial effects of CS on the growth and alleviation of biotic and/or abiotic stresses of plants^24^,^25^,^26^,^27^. In the present study, the dry weight of grain amaranths significantly increased and reached a maximum value when CS was added at the rate of 1.65 g/kg (Fig. 1). However, its rational utilization involving maximum efficiency of resources is of great importance^24^. The biomass of the grain amaranths did not decrease at the rate of 3.31 g/kg CS, revealing that the promoting effect of CS on the growth of grain amaranths might have reached a maximum critical value.

Leaf chlorophyll contents provide valuable information about photosynthesis intensity and physiological status of plants, whereas Cd accumulation in plants may decrease pigment content and restrain photosynthesis of plants^28^,^29^. Application of CS has been shown to enhance the chlorophyll content of grain amaranths cultivated in a Cd contaminated soil (Fig. 2). Similar findings have been found in the study of Bokhtiar *et al*.^24^ who observed positive effects of CS fertilizer on photosynthesis, respiration and the growth of sugarcane. However, chlorophyll contents of grain amaranths returned to the same level as those of untreated control when CS was added at a high concentration (3.31 g Si/kg Soil, CS4 treatment), indicating an optimal application amount of CS for maximum plants growth efficiency may exist between the rate of 1.65 and 3.31 g/kg CS. There is increasing evidence that silicon can mitigate the heavy metals damage in photosynthetic system of plants such as barley^30^, cucumber^31^ and cotton^32^. Silicate addition was reported to increase phosphate desorption in soil and increase plants uptake of phosphate^33^ which contributes to pigments biosynthesis^34^. Simultaneously, calcium (Ca) is an important factor affecting photosythesis process. Calcium is a structural component of photsystem II and is closely linked to photosynthesis through moderating processes such as Calvin Benson-Bassham cycle^35^. Cadmium-induced photosynthetic system damage was reported to be rehabilitated by exogenous Ca as well^36^,^37^. It was suggested that exogenous application of Ca alleviate Cd-induced impairment in plant parts by increasing membrane integrity and re-stabilized Ca-mediated signalling inside the plants^38^.

It has been proven that proper CS supplies can reduce Cd accumulation in plants^39^. The beneficial effects of silicate on reducing heavy metals uptake by plants has been first attributed to the effects of silicate ion in soil and Si accumulated in plants^7^,^40^. Application of CS significantly increased soil pH and reduced available Cd in soil (see Supplementary Fig. S1). Silicate ion is one of the important factors as it is the critical ion in soil pH regulation^24^. Moreover, the heavy metal immobilizing effect of CS has been attributed to the ability of silicate to transform heavy metals from soluble form into insoluble metal compounds of silicate^41^,^42^. In plants, it was suggested that Si deposition might cause the thickening of the cell walls of epidermis, endodermis (Casparian strips) and vascular cylinder, resulting in the restriction of apoplasmic transport of Cd^43^. However, our previous study found that higher Si in plants did not always result in lower plant Cd concentrations^10^. Silicon in plants may play a major role in alleviating metals stress and achieving cell detoxification^10^,^42^. Besides, available mineral nutrients (Ca, Mg, K, Cu, Zn)/available Cd concentration ratios were increased with increasing CS addition, especially for available Ca/available Cd ratio (see Supplementary Table S1), implying relatively higher concentrations of cations into soil solution to compete with Cd and reduce Cd competitiveness on root surface for plant uptake due to soil pH change and introduction of exogenous Ca^13^. However, how CS might modify Cd distribution and detoxification in plants remains uncertain.

Subcellular localization can help with the understanding of the mechanisms of heavy metals accumulation, transport and detoxification in plants^16^. After uptake by the plants, heavy metal detoxification is achieved by chelation and sequestration by organo-ligands at a subcellular level^44^. The function of vacuoles in metal compartmentalization and the affinity of cell walls for heavy metals play an important role in metal detoxification and tolerance of plants^45^,^46^. In this study, most of the Cd was localized at cell wall and soluble fractions (Table 1), which was consistent with the results of most recent studies. In barleys^47^, pokeweed^20^ and pea plants^45^, the largest ratio of Cd was accumulated in the soluble fraction, followed by the cell wall fraction, while maize^45^, ramie^16^ and alfalfa^48^ accumulated more Cd in the cell wall compartment than in the soluble fraction. The differences of Cd subcellular distribution between plants may be due to the distinct Cd treatment levels in different experiments and also to the variation of Cd tolerance of different plants^49^.

An important finding in this study is that an alteration of Cd subcellular distribution in grain amaranths was resulted from the application of CS, revealing an important mechanism of CS amelioration of metal stress in plants (Table 1). Cell wall is the first protective barrier to prevent invasion of heavy metals into cell^50^, followed by plasma membrane which is the first “living” structure that is target of heavy metals toxicity^51^. In the present study, the proportion of the cell wall-bound Cd in leaves of grain amaranths was reduced due to various CS treatments, whereas there was no percentage change of Cd in membrane and organelle containing fraction in leaves and in all fractions of roots (Table 1), which may indicate a restriction on Cd transportation from root to shoot. Silicon is not only a cell wall incrustation responsible for the rigidity of cells in leaves of monocots, but also found to precipitate in dicots and involved in cation-binding capacity of cell wall^52^,^53^. More importantly, calcium introduced by CS addition play an imperative role in controlling membrane structural integrity and translocation of available heavy metals into cell^54^. Calcium can compete with Cd on absorption sites and reduce negativity of the cell surface, therefore, Ca^2+^ supplementation may reduce Cd uptake via Ca channels^55^. Studies have shown that either Si or Ca^2+^ application reduced Cd-induced oxidative damage by lowering formation of ROS and improved stress resistance of Cd-stressed plants by enhancing antioxidant enzyme activity^9^,^54^,^56^,^57^. Thus, a combined effect of silicate ion and Ca^2+^ due to CS addition may promote greater efficiency in amelioration of Cd stress in plants.

An increased percentage of Cd was located in the soluble fraction in leaves of grain amaranths (Table 1). This result may be associated with the increasing coprecipitation of Cd and Si in vacuole and cytoplasm. Formation of metal silicate compound in cytoplasm and vacuoles in leaf cells is regarded as part of the heavy metals tolerance mechanism of some species, for examples, maize^42^ and *Cardaminopsis*^52^. On the other hand, calcium was shown to be involved in Cd exclusion and/or intracellular sequestration of plants by forming crystals containing high amounts of Cd and Ca^55^. More interestingly, higher percentages of Cd in chloroplast (in leaves) and trophoplast (in roots) were found in CS treated amaranths. Nwugo *et al*.^58^ investigated the mechanisms involved in Si-induced Cd tolerance in rice leaves on the molecular level, which was found to be a significant enhancement of the expression of photosynthesis and carbon dioxide assimilation associated proteins. Calcium also has a crucial role in moderating chloroplast metabolism directly or via Ca-binding protein CaM^35^. Hence, application of CS may activate a number of protein kinases and other proteins, thus to elevate the binding of Cd to organic or inorganic ligands in chloroplast and trophoplast, which is the site of plant photosynthesis and the storage site of assimilation products, respectively.

Different chemical forms of heavy metals are closely related to different biological functions, which have distinct bioavailability and toxicities. Water-soluble Cd in the inorganic form (extracted by 80% ethanol) and the organic form (extracted by deionized water) migrate more easily and are more toxic to plant cells than pectate and protein integrated Cd (1 M NaCl extractable Cd), insoluble Cd phosphate complexes (2% HAC extractable Cd) and Cd oxalate (0.6 M HCl extractable Cd) with little or no toxicity to plants.

In the present study, Cd extracted by 1 M NaCl was the predominant chemical form in grain amaranths (Table 2). These results were consistent with the previous studies in *Bechmeria nivea* L.^16^ and *Medicago sativa* L.^48^, where the largest proportion of Cd was bound in peptides and protein integrated forms (extracted by 1 M NaCl). Proteins rich in thiol and or cysteine have high binding affinity for heavy metals, especially Cd^59^,^60^. Therefore, the greater fraction of total Cd extracted by NaCl may be indicative of plant adaptation to Cd stress^61^.

Application of CS also improved Cd tolerance of grain amaranths by converting Cd into inactive forms. In the present study, there was a significant decrease in the proportion of water soluble Cd accompanied by a notable increase in the percentage of inactive forms of Cd in the subcellular fractions of both roots and shoots of CS-treated amaranths compared to untreated controls (Table 2). Specifically, a higher proportion of NaCl extractable Cd was found in CS treated plants, indicating a larger proportion of Cd integrated with pectates and proteins. It was assumed that the difference in the chemical form of Cd present in the plant cells could be relevant to the difference in Cd-induced metal-binding proteins (peptides), including phytochelatins^47^. Silicon was proven to stimulate the response of plants to heavy metals toxicity at the gene expression level. For example, higher expression levels of metallothionein (MTs) and phytochelatins (PCs) genes closely related to metals detoxification were found in Cu-stressed *Arabidopsis thaliana* treated with Si than in plants only provided with toxic levels of Cu^62^. However, these detoxification mechanisms were not merely associated with the effect of silicon in this study, but also with the influence of Ca^2+^ uptake by plants due to CS addition. As it was reported by the study of Srivastava^63^, both of the Cd^2+^ + Ca^2+^ and Cd^2+^ + Si treatments stimulated similar increasement of non-protein thiols and reduction in the level of GSH in rice seedlings, which might indicate synthesis of PCs and hydroxymethyl-PCs and other low-molecular-weight thiol-rich compounds that play an important role in heavy metals detoxicfication in plants. Therefore, application of CS may help to reduce the toxicity of Cd in plants by stimulating relatively higher proportions of peptides and proteins that can easily chelate with Cd and protect against Cd toxicity, such as MTs and PCs that are known as Cd complexers.

## Conclusion

In conclusion, application of calcium silicate (CS) mitigated the Cd toxicity of grain amaranths. The growth of grain amaranths in a Cd contaminated soil was significantly enhanced with the increasing doses of CS treatment. Photosynthesis capacity was also improved substantially, as increasing pigments contents was found in leaves of Cd stressed plants treated with silicate. Cadmium concentrations in roots and shoots of grain amaranths were significantly reduced due to silicate addition, as compared to the control. Meanwhile, the findings of subcellular distribution and chemical forms of Cd in plants provided new insights into the role of silicate in alleviating Cd toxicity and bioavailability in plants. The subcellular distribution of Cd in grain amaranths was changed, which may reveal an important mechanism of CS in amelioration of metal stress through metals compartmentation in plants. The results of chemical forms of Cd showed a significant decrease in the proportion of water soluble Cd whereas a notable increase in the percentage of pectate and protein integrated Cd, further indicated the effects of CS on reducing Cd toxicity in plants by converting Cd into inactive form from toxic free Cd iron. These results provided evidence for the involvement of CS as a specific mean for Cd immobilization and detoxification. We suggested that soil addition of calcium silicate at the amount of 1.65–3.31 g/kg CS could be an effective mean to immobilize Cd in soil and reduce crop Cd uptake.

## Materials and Methods

### Pot experiment design

A pot experiment was carried out in a green–house of South China Botanical Garden. The soil was collected from the surface layer (0–20 cm) of a vegetable garden, near a waste landfill site in the suburb of Guangzhou, China. The soil was air-dried, crushed, mixed thoroughly and passed a 1 cm mesh sieve. Chemical properties of the soil were: soil pH 6.3, organic matter 4.7%, cation exchange capacity (CEC) 13 cmol kg^−1^, available Si 82 mg kg^−1^, available P 122 mg kg^−1^ and total Cd 6.1 mg kg^−1^.Seven and a half kilogram of soil was put into each of the plastic pots with 35 cm diameter and 20 cm depth and mixed well with 0.2 g/kg N as urea and 0.2 g/kg K_2_O as KNO_3_ solution. Five levels of calcium silicate with four quadruplicates were added at the amount of 0 (CS0, control), 0.41 (CS1), 0.83 (CS2), 1.65 (CS3), 3.31 (CS4) g/kg. Each pot filled with pretreated soil was then watered with tap water daily to keep soil moisture at approximately 95% water holding capacity for one week. After soil incubation, twenty seeds of grain amaranths (*Amaranthus hypochondriacus* L. Cv. ‘K112’) were initially sowed to each pot and later shinned to six uniform seedlings (2 cm high). All pots were watered to keep soil water holding capacity at a level of about 80% during the period of plant growth. Sixty days after sowing, plants were harvested and divided into leaves, stems and roots, rinsed with distilled water. The samples were oven-dried for 72 h at 70 °C to constant weight.

### Analysis of plants and soils

Dry plant samples were ground to pass a 100 mesh sieve. After digestion of the samples using HNO_3_-HClO_4_ (4:1), Cd concentrations were analyzed using flame atomic absorption spectrometry (FAAS, HitachiZ-5300). To ensure the quality of analytical procedures, a national standard plant material (poplar leaf GBW07604) was used and blanks were also included in the digestion batches. Soil pH values were measured using a pH meter (Mettler Toledo FE20) with a water-solid ratio of 2.5:1, while available soil Cd was determined using 0.01 M CaCl_2_ solution on the day before sowing^64^.

### Measurement of chlorophyll contents

Chlorophyll a (Chl a), chlorophyll b (Chl b), total chlorophyll (Chl (a + b) and carotenoids (Car) were determined spectrophytometrically according to the method of Aron^65^. Fresh leaf discs (0.2 g, diameter 7 mm) were removed from the upper fifth leaves of grain amaranths and extracted with 10 ml of 80% (v/v) acetone in the dark for 72 h until they were blanched. The absorbance values for 663 nm, 645 nm and 470 nm wavelengths of the extracts were determined using an ultroviolet-visible spectrometer. Chl a, b, Chl (a + b) and Car were calculated using the following equations:


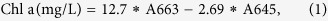



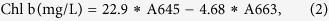



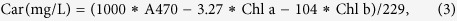



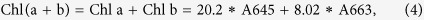


### Extraction of Cd subcellular distribution

Cells were separated into four fractions using differential centrifugation technique as suggested by Wu *et al*.^47^. Briefly, frozen leaf and root samples were prepared and homogenized in pre-cold extraction buffer (50 mM Tris–HCl, 250 mM sucrose, 1.0 mM DTE (C_4_H_10_O_2_S_2_, Sigma D8255), 5.0 mM ascorbic acid and 1.0% w-v Polyclar AT PVPP, pH 7.5). The homogenate was sieved through a nylon mesh (240 μm) and the residue was designated as fraction (FI), mainly containing cell walls and cell wall debris. The filtrate underwent a centrifugation at 1500 × g for 10 min (root sample, 2500 × g for 20 min) and the pellet retained was chloroplast-shoot/trophoplast-root containing fraction (FII). The suspension was then centrifuged at 15000×g for 35 min. The resulting precipitation and supernatant constituted the membrane and organelle containing fraction (FIII) and soluble fraction (FIV), respectively. All steps were carried out at 4 °C. Each fraction was oven dried and then treated with wet digestion process. Cadmium concentrations in different fractions were analyzed by flame atomic absorption spectrometry (FAAS, Hitachi Z-5300).

### Extraction of Cd in chemical forms

Determination of Cd chemical forms was carried out by designated extraction solutions listed below^66^: (1) 80% ethanol, extracting inorganic Cd including nitrate/nitrite, chloride, and aminophenol cadmium. (2) Distilled water (d-H_2_O), extracting water soluble Cd of organic acid and Cd(H_2_PO_4_)_2_. (3) 1 M NaCl, extracting Cd integrated with pectates and protein. (4) 2% acetic acid (HAc), extracting insoluble CdHPO_4_, Cd_3_(PO_4_)_2_ and other Cd phosphate complexes. (5) 0.6 MHCl, extracting Cd oxalate. Frozen leaf and root tissues (about 2 g) were homogenized in 37.5 ml of the first extraction solution using a glass tissue homogenizer. Then the homogenate was shaken for 18 h at 30 °C, centrifuged at 5000 g for 10 min, and the first supernatant was collected in a glass beaker. The sediment was re-suspended three times with the same amount of extraction solution and shaken 2 h at 30 °C, centrifuged at 5000 g for 10 min. Then a total of 150 ml extraction solution was gathered and designated as fraction (i). The residues were subjected to the next extraction and underwent centrifugation with the same procedures mentioned above. The final sediment was defined as the residual form (fraction (vi)). Each fraction was evaporated on an electric plate at 70 °C to dryness and digested with an acid oxidative mixture of HNO_3_: HClO_4_ (3:1, v/v).

### Statistical analysis

Data from plant and soil samples were statistically analyzed using one-way ANOVA at a significance level of *p* < 0.05 using SPSS 11.6 software. Duncan’s new multiple range test was used to detect any significant differences between means of different treatments. Simple correlation analysis and linear regression analysis were used to test the relation between soil pH and available Cd.

## Additional Information

**How to cite this article:** Lu, H. *et al*. Influences of calcium silicate on chemical forms and subcellular distribution of cadmium in *Amaranthus hypochondriacus* L. *Sci. Rep.* **7**, 40583; doi: 10.1038/srep40583 (2017).

**Publisher's note:** Springer Nature remains neutral with regard to jurisdictional claims in published maps and institutional affiliations.

## Supplementary Material

Supplementary Information

## Figures and Tables

**Table 1:** Subcellular distribution of Cd in in the roots and leaves of grain amaranths (*Amaranthus hypochondriacus* L.) grown in a Cd contaminated soil under different calcium silicate treatments (mg/kg, DW).

Tissue	Treatments	Cd content							
		FI		FII		FIII		FIV	
Leaf	CS0	44 ± 5.0 a	(39 ± 3.1 A)	7.3 ± 0.88 a	(6.4 ± 0.85 B)	3.0 ± 0.48 a	(2.7 ± 0.48 B)	59 ± 3.9 ab	(52 ± 3.0 B)
	CS1	25 ± 1.6 b	(26 ± 1.4 B)	6.7 ± 1.2 a	(6.7 ± 0.87 B)	3.3 ± 0.20 a	(3.4 ± 0.25 B)	64 ± 6.5a	(64 ± 1.1 A)
	CS2	24 ± 1.3 b	(29 ± 1.4 B)	8.1 ± 0.74 a	(9.4 ± 0.85 AB)	2.2 ± 0.24 b	(2.6 ± 0.29 B)	51 ± 1.9 b	(59 ± 0.75 A)
	CS3	8.1 ± 1.1 c	(24 ± 2.6 B)	3.0 ± 0.07 b	(8.9 ± 0.48 B)	1.4 ± 0.12 b	(4.2 ± 0.25 A)	21 ± 0.80 c	(63 ± 2.5 A)
	CS4	2.8 ± 0.44 c	(27 ± 3.1 B)	1.2 ± 0.17 b	(12 ± 1.8 A)	0.27 ± 0.01 c	(2.7 ± 0.25 B)	5.8 ± 0.39 d	(58 ± 2.6 AB)
Root	CS0	26 ± 1.5 a	(44 ± 1.9 A)	9.9 ± 0.65 a	(17 ± 1.3 A)	4.0 ± 0.24 a	(6.7 ± 0.63 A)	19 ± 0.95 a	(33 ± 1.2 A)
	CS1	21 ± 3.2 a	(41 ± 2.9 A)	7.9 ± 0.70 ab	(16 ± 1.1 A)	3.0 ± 0.43 a	(6.0 ± 0.63 A)	18 ± 1.5 a	(37 ± 1.9 A)
	CS2	18 ± 3.7 a	(38 ± 3.3 A)	8.9 ± 2.1 a	(18 ± 1.4 A)	3.3 ± 0.54 a	(7.0 ± 0.95 A)	17 ± 2.2 a	(36 ± 3.5 A)
	CS3	11 ± 1.5 b	(43 ± 4.8 A)	4.6 ± 0.95 b	(18 ± 2.4 A)	1.8 ± 0.28 b	(7.1 ± 0.85 A)	8.3 ± 1.2 b	(32 ± 3.0 A)
	CS4	8.3 ± 0.70 b	(44 ± 1.6 A)	4.3 ± 0.92 b	(22 ± 38 A)	1.2 ± 0.19 b	(6.3 ± 0.75 A)	5.4 ± 0.70 b	(29 ± 3.7 A)

CS0, CS1, CS2, CS3 and CS4 represent treatments that calcium silicate was added at the amount of 0, 0.41, 0. 83, 1.65 and 3.31 g/kg, respectively. FI: cell wall fraction; FII: chloraplast in leaf or trophoplast in root; FIII: membrane and organelle containing fraction; FIV: soluble fraction. Data in the table are expressed as mean ± SE of the quadruplicates. Means in each column with the same letter are not significantly different at *P* < 0.05. Data in parentheses indicate the Cd proportion (%) in each fraction to the total Cd content in all fractions.

**Table 2:** Concentrations of different chemical forms of Cd in the roots and leaves of grain amaranths (*Amaranthus hypochondriacus* L.) under different calcium silicate treatments (mg/kg, DW).

Tissue	Treatments	Cd content											
		F(i)		F(ii)		F(iii)		F(iv)		F(v)		F(vi)	
Leaf	CS0	2.3 ± 0.25 a	(2.6 ± 0.46 B)	19 ± 2.6 a	(21 ± 1.9 A)	42 ± 5.2 a	(46 ± 3.0 C)	25 ± 1.6 a	(28 ± 3.4 A)	1.3 ± 0.08 a	(1.5 ± 0.04 B)	0.004 ± 0.000 bc	(0.005 ± 0.0004 C)
	CS1	1.7 ± 0.11 a	(2.0 ± 0.12 B)	17 ± 1.7 a	(20 ± 1.6 A)	48 ± 4.2 a	(56 ± 3.5 B)	18 ± 1.4 b	(21 ± 1.8 BC)	1.0 ± 0.12 ab	(1.2 ± 0.14 B)	0.003 ± 0.000 c	(0.003 ± 0.0005 C)
	CS2	1.9 ± 0.27 a	(2.7 ± 0.33 B)	15 ± 0.90 a	(20 ± 1.1 A)	40 ± 3.6 a	(54 ± 2.1 B)	16 ± 2.4 b	(22 ± 2.5 ABC)	0.94 ± 0.12 b	(1.3 ± 0.15 B)	0.007 ± 0.001 a	(0.01 ± 0.001 C)
	CS3	1.8 ± 0.25 a	(6.1 ± 0.54 A)	1.9 ± 0.21 b	(6.7 ± 0.19 B)	17 ± 1.6 b	(57 ± 1.3 B)	8.1 ± 1.0 c	(28 ± 1.7 AB)	0.59 ±0.10 c	(2.0 ± 0.28 A)	0.006 ± 0.001 ab	(0.02 ± 0.002 B)
	CS4	0.54 ± 0.03 b	(6.5 ± 0.44 A)	0.41 ± 0.03 b	(5.0 ± 0.44 B)	5.7 ± 0.33 c	(67 ± 0.85 A)	1.6 ± 0.12 d	(19 ± 0.7 C)	0.17 ± 0.004 d	(2.1 ± 0.10 A)	0.003 ± 0.000 c	(0.03 ± 0.004 A)
Root	CS0	2.2 ± 0.16 a	(3.0 ± 0.31 BC)	12 ± 2.3 a	(15 ± 1.8 A)	62 ± 8.2 a	(79 ± 2.2 B)	2.0 ± 0.18 a	(2.6 ± 0.28 BC)	0.36 ± 0.13 a	(0.48 ± 0.13 AB)	0.02 ± 0.004 a	(0.03 ± 0.003 C)
	CS1	1.4 ± 0.07 b	(2.6 ± 0.21 C)	7.9 ± 0.66 b	(14 ± 0.80 A)	47 ± 6.3 ab	(81 ± 1.1 AB)	1.1 ± 0.18 bc	(1.9 ± 0.17 C)	0.17 ± 0.03 b	(0.31 ± 0.06 B)	0.02 ± 0.003 a	(0.05 ± 0.003 BC)
	CS2	1.8 ± 0.27 ab	(3.4 ± 0.47 B)	7.1 ± 1.1 b	(13 ± 1.3 A)	45 ± 5.8 ab	(81 ± 1.6 AB)	1.3 ± 0.15 b	(2.5 ± 0.24 BC)	0.13 ± 0.02 b	(0.26 ± 0.04 B)	0.02 ± 0.007 a	(0.05 ± 0.003 BC)
	CS3	1.7 ± 0.05 b	(4.8 ± 0.52 A)	3.4 ± 0.33 c	(9.3 ± 0.30 B)	30 ± 4.0 bc	(82 ± 1.1 AB)	1.4 ± 0.19 b	(3.8 ± 0.46 A)	0.16 ± 0.05 b	(0.43 ± 0.07 AB)	0.03 ± 0.008 a	(0.07 ± 0.01 AB)
	CS4	0.79 ± 0.08 c	(3.8 ± 0.08 AB)	1.7 ± 0.08 c	(8.6 ± 0.68 B)	17 ± 1.6 c	(84 ± 0.55 A)	0.62 ± 0.09 c	(3.0 ± 0.21 AB)	0.13 ± 0.01 b	(0.65 ± 0.08 A)	0.02 ± 0.003 a	(0.08 ± 0.01 A)

CS0, CS1, CS2, CS3 and CS4 represent treatments that calcium silicate was added at the amount of 0, 0.41, 0. 83, 1.65 and 3.31 g/kg, respectively. F (i): inorganic Cd extracted by 80% ethanol; F (ii): water soluble Cd extracted by deionized water; F (iii): pectate- and protein- integrated Cd extracted by NaCl; F (iv): phosphate Cd extracted by 2% HAc; F (v): oxalate Cd extracted by 0.6 M HCl; F (vi): Cd in residual form. Data in the table are expressed as mean ± SE of the quadruplicates. Means in each column with the same letter are not significantly different at *P* < 0.05. Data in parentheses indicate the Cd proportion (%)in each fraction to the total Cd content in all fractions.
