# Supplementary material for: Influences of calcium silicate on chemical forms and subcellular distribution of cadmium in Amaranthus hypochondriacus L
Source: Sci Rep. 2017 Jan 11;7:40583. doi: 10.1038/srep40583 (PMC5225445; doi:10.1038/srep40583)
Supplement: Supplementary Information [file srep40583-s1.doc]

**Title:** Influences of calcium silicate on chemical forms and subcellular distribution of cadmium in *Amaranthus hypochondriacus* L.

**Authors:**

Huanping Lu, Zhian Li^*^, Jingtao Wu, Yong Shen, Yingwen Li, Bi Zou, Yetao Tang, Ping Zhuang^*^

**MS NO.: SREP-16-19077**

**Supplementary Information:**

**Supplementary Figure S1. Soil available Cd and soil pH under different treatments.** CS0, CS1, CS2, CS3 and CS4 represent treatments that calcium silicate was added at the amount of 0, 0.41, 0.83, 1.65 and 3.31 g/kg, respectively. Soil pH values were measured using a pH meter (Mettler Toledo FE20) with a water-solid ratio of 2.5:1. Available soil Cd was determined using 0.01 M CaCl_2_ solution on the day before sowing. Error bars represent +/- SE of the quadruplicates.

**Supplementary Table S1 Concentration ratios of available Ca, Mg, K, Cu, Zn, Mn to available Cd in soil**

| Treatments | Ca | Mg | K | Cu | Zn | Mn |
| --- | --- | --- | --- | --- | --- | --- |
| CS0 | 1928±85 c | 30±2.2 c | 151±13 c | 9.0±0.37 c | 81±3.1 d | 2.5±0.13 b |
| CS1 | 2641±86 c | 34±1.1 c | 165±6.1 c | 11±0.38 c | 94±1.6 cd | 1.7±0.16 c |
| CS2 | 3745±179 c | 39±1.9 c | 183±11 c | 13±0.18 c | 120±5.7 c | 1.1±0.07 d |
| CS3 | 9047±351 b | 61±3.6 b | 281±36 b | 26±1.23 b | 231±11 b | 1.1±0.1 d |
| CS4 | 26120±2321 a | 134±12 a | 668±36 a | 56±4.8 a | 516±17 a | 5.2±0.17 a |

CS0, CS1, CS2, CS3 and CS4 represent treatments that calcium silicate was added at the amount of 0, 0.41, 0.83, 1.65 and 3.31 g/kg, respectively. Available Ca, Mg and K were extracted by 1 M [CH](http://dict.youdao.com/w/ammonium acetate/" \l "keyfrom=E2Ctranslation)_3_COONH_4_ solution. Available Cu, Zn and Mn was extracted by 0.1 M HCl solution. Data in the table represented the concentration ratios of available Ca, Mg, K, Cu, Zn, Mn to available Cd in soil and expressed as mean ± SE of the quadruplicates. The means with the same letter in each column are not significant different (*p* > 0.05).
